# Mr. Bellot, in Reply to Mr. Bradley

**Published:** 1808-09

**Authors:** Abraham Bellot

**Affiliations:** Oldham, Lancashire


### *To the Editors of the Medical and Physical Journal.*

GENTLEMEN,

WHEN such a considerable diversity of opinion has prevailed amongst anatomists and surgeons, concerning the operation for crural hernia, with respect to the *part* of the arch which ought be divided, the *direction* in which the division ought to be made, and the *manner* of executing it, we can scarcely say as yet that there is any established rule. In my operation for crural hernia, though I may seem to Mr. Bradley to have deviated from established rule, I hope, by a little explanation, it will appear, that I have scarcely deviated from what he calls the general rule; or, if at all, it has been on the safe side. In crural hernia, when the stricture is owing to the liga-

 ment ment only, the difficulty of introducing the finger must necessarily be very great; but when the stricture (as it was in this case) is at the neck of the sac, the finger then may be got under the arch with more facility, cutting gently with the bistoury upon the tip of the finger; in some instances the arch has been found remarkably wide. Le Dran mentions a case, where one might almost thrust four fingers with the integuments underneath it. Obs. de Chirurg. T. 2, Obs. 58.

The necessity of dividing the ligaments will appear, in order to separate the adhesions round the neck of the sac, and to get above the mouth of it, as no opening could safely be made below, from the adhesion which had taken place. If Mr. Bradley will look at the patient placed for the operation, I think he will then allow, that we may cut obliquely downwards, and towards the pubis. The incision was first begun nearly in a direction towards the umbilicus, then turning the edge of the bistoury obliquely towards the pubis, carrying it downwards, in order to avoid the spermatic end. Dr. Hull states, that in his observations on crural hernia, that the spermatic artery passes along the upper and inner side of the neck of the sac, and is liable to be divided if the ring be cut upwards and inwards; but to avoid this, he recommends "a small incision to be made through the tendon of the oblique externus, rather more than half an inch above the anterior margin of Poupart's ligament, dividing the tendon upon the direction down to the anterior margin; the spermatic cord is then to be drawn up, and the ligament divided upwards and inwards." This, certainly, would be a good general rule to follow.

Mr Cooper says, "that a stricture is a circle produced in the same way as if a cord were tied round the protruded parts, and that a division might be made at any part except the posterior, where the bone is placed, if other circumstances did not prevent it." Surely then, there can be no impropriety in speaking of adhesion being torn asunder all round, not dissected, for here the knife would be inadmissible. After the division of the ligament and the adhesions were separated, the whole of the sac with its contents might have been returned, without opening the sac; but we might as well have performed no operation at all, as to have returned the whole when the stricture was at the neck of the sac, with the contents adhering. The adhesions prevented the contents being pushed back, and also from getting into the sac below its neck; when this this is the case, Monro recommends the tumour to be dragged down to get it into the sac from above downwards, which was done. Mr. B. cannot deny the elasticity of the peritoneum; otherwise, how does the hernia first take place. The tumour was certainly dragged down, but not perhaps to the extent of *half an inch or more*, which Mr. B. thinks would be necessary, to expose the neck of the sac: he will recollect, the ligament was before divided; notwithstanding, he seems to doubt its ever being cut at all. This division, together with stretching the sac, brought the neck into view. The possibility of breaking adhesions, with success, is evident from the result of the case: this was effected by keeping the probe towards the sac, and not rudely pushing forwards, regardless whether the intestine is perforated or not. The opening which was made above the mouth of the sac, was not made into the *cavity of the belly*; for every portion of peritoneum which appears, or is made to appear, on the outside of the crural ring, must still be sac. Mr. Cooper relates a case, where symptoms of strangulation had existed for 10 days, and in 15 minutes after the reduction, an evacuation took place. Vid. Cooper's Hern. part ii, p. 8. In my case, the operation was performed on the 11th day, and in two hours after, an evacuation took place: this, therefore, does not need to surprise Mr. B. It would likewise be unnecessary to give a detail of symptoms, when none occurred that were particularly unpleasant, after the operation. I believe I am warranted in separating the adhesions, by the opinion of Mr. Lawrence, as well as from the success with which it has been attended. Mr. B. seems to have fallen into an error, when he says, "and when at the same time the intestine is much indurated." Now, it was not the intestine that was indurated, but the omentum. I am not aware of having made any particular innovations. The operation for crural hernia is always formidable; and in this case, more than ordinary difficulties had to be encountered; it therefore gives me great pleasure to say, the person is still a living witness of its complete success.

I am, &c.

ABRAHAM BELLOT.

*Oldham, Lancashire, Aug.* 11, 1808.

